# Effects of graft materials on bone regeneration using the osseous shell technique: an experimental study in rats

**DOI:** 10.1186/s40729-026-00693-3

**Published:** 2026-05-18

**Authors:** Nyungkwang Kwon, Kazuhiro Imoto, Yuta Yanagisawa, Reina Minohara, Atsuya Abe, Shinnosuke Nogami, Shinsuke Ohba, Kensuke Yamauchi

**Affiliations:** 1 Division of Oral and Maxillofacial Reconstructive Surgery, Department of Disease Management Dentistry, Tohoku University Graduate School of Dentistry, 4-1 Seiryo-machi, Aoba-ku, Sendai, Miyagi 980-8575 Japan; 2 Department of Tissue and Developmental Biology, Graduate School of Dentistry, Osaka University, Suita, Osaka Japan

**Keywords:** Bone augmentation, Bone grafting, Shell technique, Rat mandibular model

## Abstract

**Background:**

Autogenous bone (AB) is considered the gold standard for alveolar bone grafting, but its limitations have prompted the development of synthetic alternatives. The Shell technique provides a stable framework for bone augmentation. However, few studies have directly compared different graft materials under intraoral-like conditions, especially in mandibular models. This study aimed to quantitatively compare the osteogenic capacity of four graft materials—autogenous bone (AB), β-tricalcium phosphate (β-TCP), octacalcium phosphate collagen composite (OCPC), and atelocollagen absorbable sponge (AAS)—using a rat mandibular shell model that simulates intraoral conditions.

**Methods:**

Cortical bone blocks were harvested from the mandibular body of 36 male Wistar rats, and mandibular reconstruction was performed using the Shell technique with cortical bone plates. Four graft materials, particulate AB, β-tricalcium phosphate (β-TCP), octacalcium phosphate collagen composite (OCPC), and Atelocollagen Absorbable Sponge (AAS), were evaluated. Each postoperative cohort consisted of 12 rats in total (*n* = 3 per group × 4 groups), and histological and histomorphometric analyses were performed at 8, 12, and 16 weeks postoperatively.

**Results:**

AB generated significantly more new bone than the other materials at all time points. OCPC induced moderate limited regeneration until later stages, while β-TCP and AAS resulted in limited bone growth. The mandibular model effectively simulated oral anatomy and provided reliable structural support throughout the procedure.

**Conclusions:**

AB showed better osteogenic capacity than synthetic and composite materials. Use of the mandibular Shell technique with a rat model of mandibular defects proved useful for evaluating bone grafts under clinically relevant conditions.

## Background

Restoration of alveolar bone volume is a prerequisite for successful and long-term stable dental implant placement. Horizontal and vertical bone deficiencies are commonly encountered in clinical settings, especially in patients with chronic periodontitis, trauma, or long-standing tooth loss. Various surgical techniques have been developed to augment deficient alveolar ridges, among which the Shell technique has gained significant attention. The Shell technique, was originally introduced by Khoury and Hanser as a method for reconstructing three-dimensional alveolar ridge defects [1]. This technique involves harvesting thin cortical bone plates, typically from the mandibular ramus, and fixing them to form a rigid outer framework, which is subsequently filled with particulate autogenous bone. The biological rationale of this approach lies in the combination of mechanical stability and space maintenance for bone regeneration, while preserving vascularization and minimizing resorption of the grafted area [2]. Clinically, the shell technique has gained widespread acceptance for both horizontal and vertical ridge augmentation, particularly in implant dentistry, due to its high predictability and its ability to reconstruct complex alveolar defects [3]. More recently, modifications of the technique, including the use of allogeneic bone plates and alternative biomaterials, have been explored to reduce donor site morbidity and expand clinical applicability, reflecting the ongoing development of this technique [4]. Autogenous bone (AB) grafts have long been considered the gold standard due to their inherent osteogenic, osteoconductive, and osteoinductive properties [5]. These biological characteristics enable autografts to actively contribute to new bone formation through cellular supply, scaffold function, and induction of host tissue responses. However, the use of such grafts has drawbacks, including donor site morbidity, increased surgical time, and limited graft volume. These limitations pose clinical challenges, especially in cases requiring large grafts.

To address these issues, alternative graft materials, such as alloplastic and composite biomaterials, have been developed and introduced. Among them, beta-tricalcium phosphate (β-TCP) exhibits good biocompatibility and biodegradability, gradually resorbing and being replaced by new bone, although it lacks intrinsic osteoinductive capacity [6]. Meanwhile, octacalcium phosphate combined with collagen composites (OCPC) show promising osteoinductive properties by mimicking early biological apatite formation [7, 8].

In addition, collagen-based matrices such as Atelocollagen Absorbable Sponge (AAS) are widely used in regenerative medicine due to their excellent biocompatibility and low immunogenicity, serving as scaffolds or carriers for cells and growth factors. However, their osteogenic potential is limited unless combined with bone particles, stem cells, or bioactive molecules [9].

Despite the growing use of such materials, few studies have directly compared their osteogenic potential under identical experimental conditions. Such comparisons among materials in models that simulate clinical bone augmentation procedures such as the Shell technique remain an important research focus. Rat models provide controlled environments to investigate the biological behaviour of graft materials in vivo, allowing detailed histological and histomorphometric analyses [10].

Therefore, in this study, we experimentally compared the osteogenic capacity of different graft materials, autogenous particulate bone, OCPC, β-TCP, and AAS matrices, using a Rat mandibular bone graft model combined with the Shell technique.

## Methods

### Animals

Ten-week-old, male, Wistar rats (*n* = 36, body weight: 250–300 g) were used. Only male rats were used in this study to minimize variability associated with hormonal fluctuations, which may influence bone metabolism and bone regeneration [11]. The animals were housed in standard cages under specific-pathogen-free conditions, maintained at a controlled temperature (22 ± 2 °C) and humidity (50–60%) with a 12-h light/dark cycle. All rats had ad libitum access to standard rodent chow and water. The protocol was approved by the Animal Care and Use Committee of Tohoku University, Sendai, Japan, in accordance with local laws and regulations (approval no. 2023DnA-014-01). Animals were randomly allocated to experimental groups using a computer-generated randomization table created with Microsoft Excel (Microsoft Corporation, Redmond, WA, USA), based on a random number function.

### Surgery

The rats were anesthetised via inhaled 5% sevoflurane (Viatris) in oxygen, followed by intraperitoneal injection of a mixed anaesthetic containing medetomidine (0.3 mg/kg), midazolam (4.0 mg/kg), and butorphanol (5.0 mg/kg). A local anaesthetic, consisting of 1.8 mL of a 2% xylocaine and epinephrine solution (1:80,000; Dentsply Sankin, Tokyo, Japan) was injected. All surgery was performed under standard sterile conditions.

**Fig. 1:**
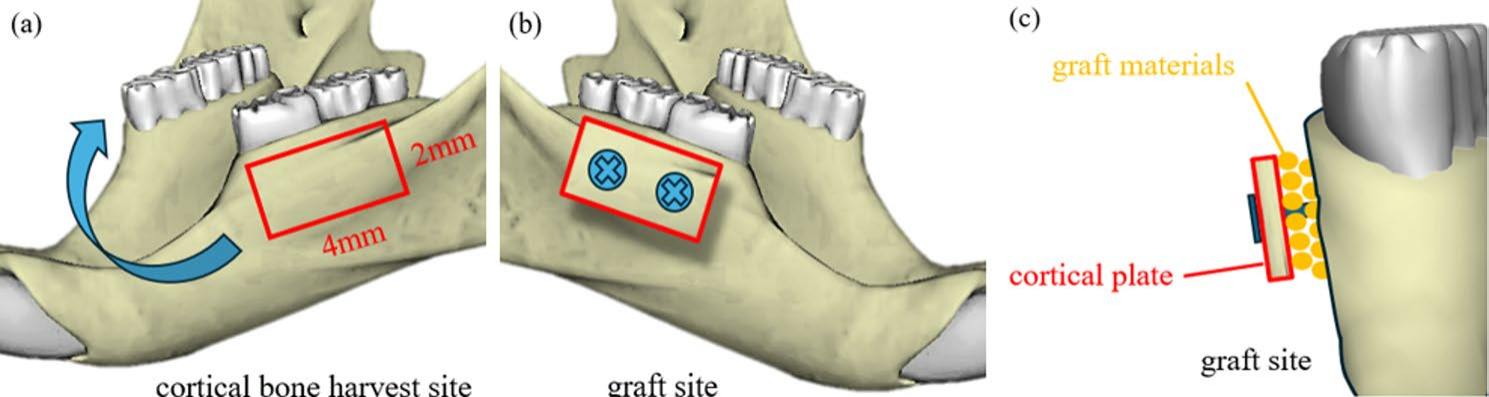
Experimental Model of the Shell Technique in the Rat Mandible, **a** A block of cortical bone was harvested from the buccal aspect of the mandibular body on one side. **b** The harvested cortical bone block was trimmed to a thin plate, positioned with a gap between it and the mandibular body, and fixed with screws. (c) After fixation, the graft material was packed into the gap

A skin incision was made in the left submandibular region, and the subcutaneous tissues were carefully dissected to expose the left mandibular body. Using an ultrasonic bone-cutting device (VarioSurg; NSK-Nakanishi Japan, Tokyo, Japan), a cortical bone block measuring 2 mm × 4 mm was harvested from the left mandibular body (Fig. 1). After haemostasis, the incision was closed in layers. Then the harvested bone block was thinned using the same ultrasonic device to reduce its thickness and adapt it as a cortical shell.

**Fig. 2:**
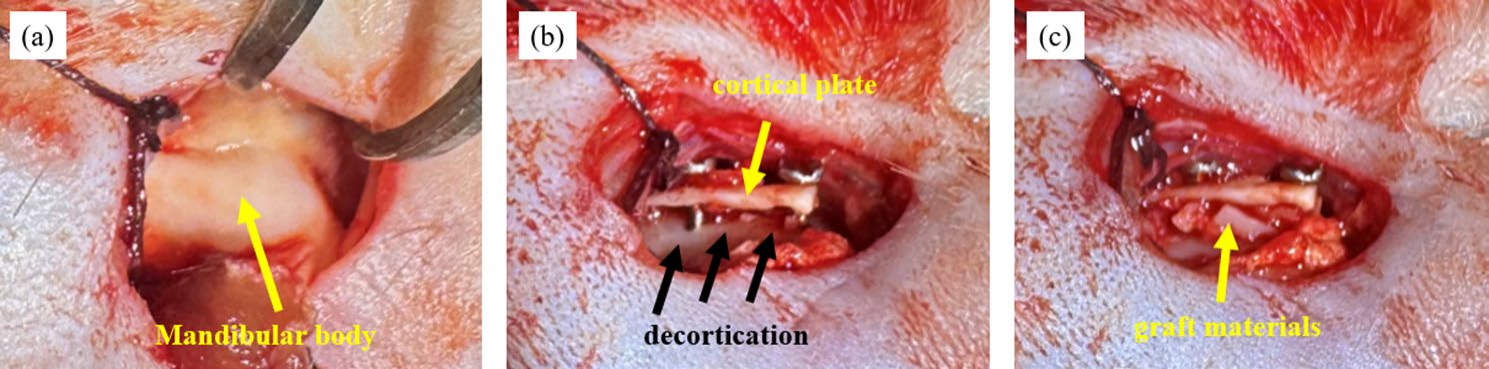
Surgical procedures on the graft side, **a** Expose the mandibular body on the graft side. **b** Perform decortication on the mandibular body and fix the cortical plate. **c** Pack the graft material into the bone gap

Subsequently, a second skin incision was made in the right submandibular region. The underlying tissues were similarly dissected to expose the right mandibular body (Figs. 1 and 2). The previously prepared cortical bone plate was fixed to the buccal surface of the mandible using a stainless-steel screw (diameter 0.6 mm, height 2.0 mm), applying the Shell technique (Figs. 1 and 2).

The space between the cortical plate and the native mandibular bone (bone gap) was filled according to four experimental groups (Fig. 2), using crushed AB harvested from the same animal (AB group; *n* = 3), octacalcium phosphate collagen composite (OCPC group; Bonarc^®^, TOYOBO; *n* = 3), β-tricalcium phosphate with a cotton-like structure (β-TCP group; ReBOSORB^®^, ORTHOREBIRTH; *n* = 3), and absorbable AAS sponge (AAS group; TERUPLUG^®^, GC; *n* = 3). Thus, each postoperative cohort consisted of 12 rats in total (*n* = 3 per group × 4 groups). All surgical procedures, including cortical bone harvesting, thinning of the cortical plate, and graft placement, were performed by the same experienced operator to ensure consistency and minimize procedural variability. At 8, 12, and 16 weeks postoperatively, one cohort (12 rats) was sacrificed, such that 36 animals were used in this study. Each time point represented an independent cohort, without repeated use of the same animals. Transcardial perfusion fixation was performed using 4% paraformaldehyde, and the mandibles were harvested for subsequent analysis. The excised mandibles were fixed in 4% paraformaldehyde for 24 h at 4 °C.

The sample size (*n* = 3 per group at each time point) was determined based on previous experimental studies using rat models for bone regeneration. In addition, in accordance with ethical guidelines for animal experimentation and the principle of reduction, the number of animals was kept to the minimum required to achieve the study objectives. This study was designed as a pilot exploratory study to evaluate biological trends rather than to establish definitive statistical conclusions.

### Tissue preparation and histological evaluation

Bone formation was evaluated via microcomputed tomography (micro-CT) (Comscantechno, Co., Ltd., Yokohama, Japan) at 90 µA and 90 kV. The voxel size was set at 10 μm, and the region of interest was standardized to the defined cortical plate-to-mandibular body gap. The extent of osteogenesis was evaluated in three dimensions. For quantitative analysis, the region of interest (ROI) was defined as the standardized space between the cortical plate and the native mandibular bone (bone gap), which was consistently applied across all specimens. Segmentation of mineralized tissue was performed using a fixed global threshold value to distinguish newly formed bone from surrounding soft tissue and residual graft materials. The threshold was determined based on preliminary calibration and applied uniformly to all specimens to ensure consistency and minimize operator-dependent bias. The volume of new bone (mm^3^) was measured using image software (TRI/3D-BON-FCS64; Ratoc System Engineering Co., Tokyo, Japan). Next, all specimens were decalcified in phosphate-buffered saline (PBS) with 10% (w/v) EDTA at 4 °C for 30 days, dehydrated in ethanol, cleared in xylene, and embedded in paraffin. Frontal sections (5 μm thickness) were prepared and mounted on glass slides. New bone in the bone gap was morphologically evaluated using hematoxylin and eosin, elastica van Gieson, and Masson staining. Investigators performing micro-CT analyses and histological evaluations were blinded to group allocation. No animals were excluded, and all specimens were analyzed.

### Statistical analysis

The volume of newly formed bone (BV), obtained from micro-CT analysis, was expressed as mean ± standard deviation (SD) for each group. Prior to statistical analysis, the normality of data distribution was assessed using the Shapiro–Wilk test, and homogeneity of variance was evaluated using Levene’s test. As these assumptions were not violated, one-way analysis of variance (ANOVA) was applied to evaluate differences in BV among the four experimental groups at each postoperative time point (PO8W, PO12W, and PO16W). When ANOVA indicated a significant difference, Tukey’s honestly significant difference post hoc test was used for multiple comparisons. If the assumptions for parametric testing had not been met, a non-parametric alternative (Kruskal–Wallis test) would have been considered. All statistical analyses were conducted using GraphPad Prism (version 10.6.0; GraphPad Software, San Diego, CA, USA) and Python (version 3.13.7; Python Software Foundation, Wilmington, DE, USA) with the statsmodels package. A p-value < 0.05 was considered statistically significant.

## Results

### Micro-CT and histomorphometric analyses

**Fig. 3:**
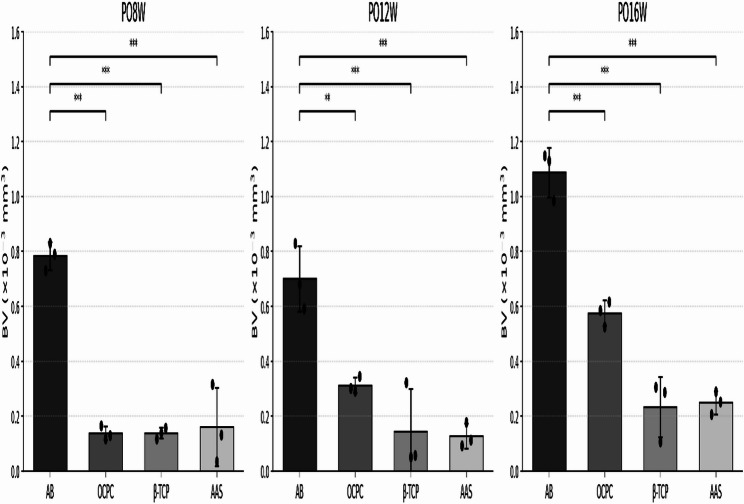
Comparison of newly formed bone volume among groups at each time point. The BV in the AB group was significantly greater than that in all other groups at all postoperative time points. In all experimental groups, the volume of newly formed bone increased with the progression of the healing period. A *p* < 0.05 was considered a significant difference. Newly formed bone volume (BV) at each postoperative time point. Bars indicate mean ± standard deviation (SD), and black horizontal markers indicate the median. Values are expressed in mm^3^

Micro-CT revealed significant differences in BV among the four experimental groups at each postoperative time point (Table 1; Fig. 3).

**Fig. 4:**
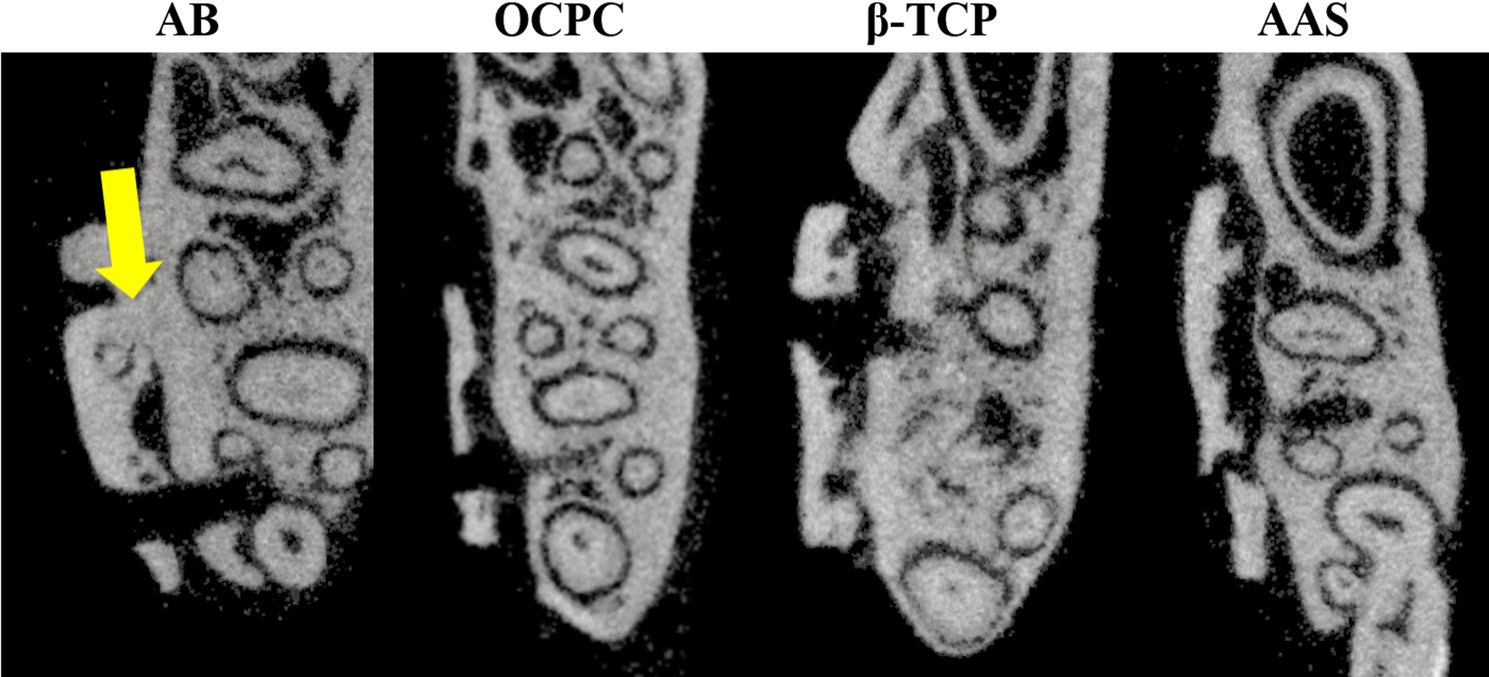
µCT images of the mandible at 16 weeks postoperatively in this study. Only in the AB group, the gap between the mandibular body and the graft plate was packed with new bone (arrows)

At PO8W, the BV in the AB group (0.784 ± 0.05 mm^3^) was significantly higher than that in the OCPC, β-TCP, and AAS groups (*p* < 0.05 for all comparisons). No significant differences were observed among the OCPC, β-TCP, and AAS groups (Fig. 4).

At PO12W, the AB group (0.701 ± 0.12 mm^3^) remained significantly higher than all other groups (*p* < 0.05). The OCPC group showed a higher BV than the β-TCP and AAS groups, although this difference was not statistically significant.

At PO16W, the AB group (1.088 ± 0.09 mm^3^) continued to show significantly greater BV than the OCPC, β-TCP, and AAS groups (*p* < 0.05). The OCPC group demonstrated a significant increase in BV compared to PO8W (*p* < 0.05), whereas no significant temporal changes were observed in the β-TCP and AAS groups.

**Table 1:** Summary of newly formed bone volume (BV)

Group	BV (mm^3^)		
	PO8W	PO12W	PO16W
AB	**0.784 ± 0.051**	**0.701 ± 0.120**	**1.088 ± 0.090**
	0.791 (0.051)	0.681 (0.237)	1.130 (0.165)
OCPC	**0.137 ± 0.025**	**0.313 ± 0.029**	**0.576 ± 0.046**
	0.129 (0.035)	0.302 (0.054)	0.586 (0.090)
β-TCP	**0.139 ± 0.020**	**0.143 ± 0.156**	**0.233 ± 0.110**
	0.141 (0.020)	0.057 (0.267)	0.287 (0.200)
AAS	**0.161 ± 0.143**	**0.128 ± 0.045**	**0.249 ± 0.042**
	0.132 (0.281)	0.114 (0.064)	0.251 (0.084)
*P*-value	1.45 × 10^−5^	4.04 × 10^−4^	2.68 × 10^−6^

Data are presented as mean ± standard deviation (SD) (upper row) and median (interquartile range, IQR) (lower row). Values are expressed in mm^3^. Micro-CT revealed distinct differences in BV among the four experimental groups at each postoperative time poin. The AB group exhibited the highest BV across all time points. Notably, the OCPC group showed a marked increase in BV between PO8W and PO16W, while the β-TCP and AAS groups exhibited minimal changes over time. A *p*-value < 0.05 was considered statistically significant

Bold values indicate mean ± standard deviation (SD), whereas non-bold values indicate median (interquartile range, IQR)

### Histological findings

#### AB group

**Fig. 5:**
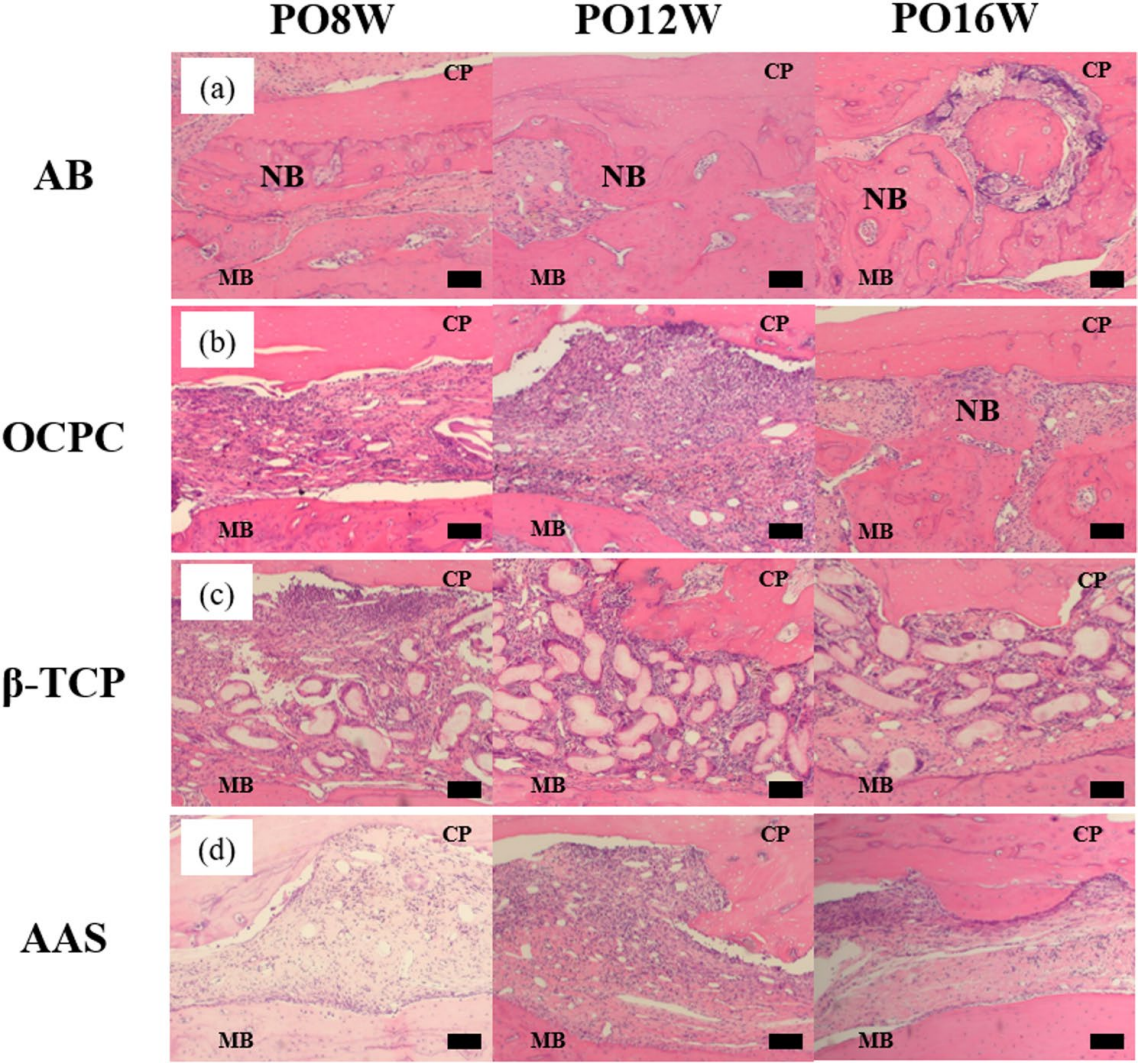
Postoperative changes in the bone gap. **a** In the AB group, new bone formation was observed from 8 weeks postoperatively. **b** In the OCPC group, new bone formation was observed at 16 weeks postoperatively. **c** In the β-TCP group, the material remained even at 16 weeks postoperatively, but new fibrous tissue formation was observed on the mandibular body side. **d** In the AAS group, the material had disappeared by 12 weeks postoperatively, and new fibrous tissue formation was observed. MB; mandibular body, CP; cortical plate, NB; newly formed bone, bar = 0.1 mm, Hematoxylin and Eosin (H&E) staining

**Fig. 6:**
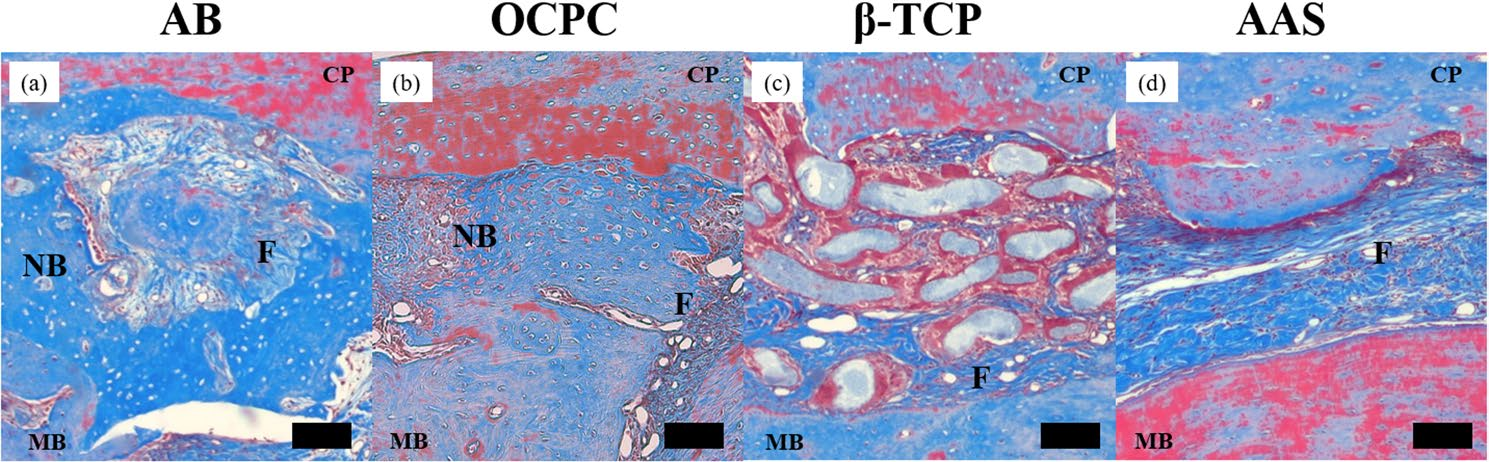
Postoperative changes in the bone gap at 16 weeks postoperatively. **a** In the AB group, Collagen fibers were observed surrounding the grafted particulate autogenous bone, which was encased by newly formed bone. **b** In the OCPC group, Collagen fibers were observed around the newly formed bone, and cells were present within the bone matrix. **c** In the β-TCP group, Collagen fibers were observed on the mandibular body side. **d** In the AAS group, Collagen fibers were observed in the bone gap. MB; mandibular body, CP; cortical plate, NB; newly formed bone, F; newly Collagen fibers, bar = 0.1 mm, Masson’s trichrome staining

At PO8W, newly formed bone was clearly observed, with trabecular structures bridging the space between the cortical shell and the host bone, indicating early maturation. By PO12W, the trabeculae had thickened, and integration with the host bone was nearly complete. At PO16W, mature lamellar bone occupied most of the defect, accompanied by active new bone formation around the grafted particulate AB (Fig. 5). Masson’s trichrome staining at PO16W revealed collagen fibres surrounding the particulate AB, in addition to the newly formed bone (Fig. 6).

#### OCPC group

At PO8W, residual graft material was still present, and little new bone formation was observed. By PO12W, the graft material was almost completely resorbed, with an increase in fibrous connective tissue. At PO16W, partial new bone formation was noted (Fig. 5). Masson’s trichrome staining demonstrated numerous cells, presumed to be osteocytes, located at the interface between collagen fibres and the newly formed bone (Fig. 6).

#### β-TCP group

At PO8W, most β-TCP granules remained intact without any evidence of new bone deposition, and the intergranular spaces were filled with fibrous tissue. At PO12W, no bone formation was observed, with abundant residual granules surrounded by fibrous tissue. At PO16W, the granules were still not resorbed, and fibrous tissue formation predominated on the mandibular body side (Fig. 5). Masson’s trichrome staining revealed collagen fibres on the mandibular body side (Fig. 6).

#### AAS group

At PO8W, residual graft material was still present, and little new bone formation was observed. By PO12W, the graft material was almost completely resorbed, with an increase in fibrous connective tissue. At PO16W, no obvious new bone was observed, but well-developed fibrous tissue was present (Fig. 5). Masson’s trichrome staining demonstrated well-developed collagen fibres (Fig. 6).

## Discussion

We investigated the osteogenic potential of four graft materials, particulate AB, β-TCP, OCPC, and AAS, using a rat model of mandibular defects in combination with the Shell technique. AB led to the most new bone, while the other three materials demonstrated varying degrees of regenerative potential.

The superior performance of AB is consistent with its well-established osteogenic, osteoinductive, and osteoconductive properties [12, 13]. The presence of viable cells, including osteoblasts and osteoprogenitors, likely contributed to the rapid formation and remodelling of new bone [14]. These findings reaffirm that AB remains the gold standard for bone grafting in clinical practice [15].

Notably, the AB group exhibited the highest BV across all time points. However, a temporary decrease in BV was observed between PO8W and PO12W, followed by an increase at PO16W. This fluctuation is likely attributable to normal remodeling dynamics, in which early woven bone is resorbed and replaced by more mature lamellar bone. Such temporal variations highlight the dynamic nature of bone regeneration and underscore the importance of evaluating multiple postoperative time points.

Among the synthetic and composite materials, the OCPC showed significantly higher bone formation compared to β-TCP and AAS. This is in line with previous studies demonstrating that octacalcium phosphate acts as a precursor to biological apatite and induces osteogenic differentiation of mesenchymal stem cells [16, 17]. The collagen matrix may enhance this effect by serving as a scaffold that supports cellular attachment and matrix deposition [18].

β-TCP showed moderate bone formation, mainly localised around the periphery of the graft particles. Although the amount of bone formation did not reach that of the OCPC group, a slow bone-regenerative response was observed in the mandibular body. This outcome reflects its osteoconductive nature without intrinsic osteoinductive capacity [19]. Although β-TCP is widely used due to its biocompatibility and resorbability, its regenerative efficacy may be limited without the addition of bioactive factors or cells [20].

AAS alone resulted in minimal bone regeneration, with the defect area predominantly occupied by fibrous tissue. This is consistent with previous studies showing that collagen-based scaffolds lack sufficient osteogenic potential unless combined with active agents or stem cells [21]. Nevertheless, its excellent biocompatibility and space-maintaining ability suggest that it may serve as a supportive scaffold in combination therapies [22].

In this study, a difference in newly formed bone volume was observed between autogenous bone and artificial materials; however, the artificial materials were used alone. Previous reports on bone augmentation have mostly employed combinations of autogenous bone and artificial materials. It is therefore considered that utilizing the osteogenic capacity of autogenous bone in combination with artificial materials may help to unlock the latent osteogenic potential of the synthetic grafts. Furthermore, the optimal mixing ratio between autogenous and artificial bone warrants further investigation [23].

This study had several limitations. First, the use of a small animal model may not fully replicate human bone biology and healing capacity, although it allows for controlled experimental conditions and molecular-level analysis [24]. Second, the observation period was relatively short and may not have been sufficient to evaluate the long-term resorption and remodelling behaviour of synthetic materials [25]. In addition, only male animals were included in this study to reduce variability related to hormonal fluctuations, particularly those associated with estrogen, which are known to influence bone metabolism and regenerative capacity. However, this approach may limit the generalizability of the findings, as sex-related differences in bone healing and remodeling have been reported. Future studies incorporating both sexes are warranted to further validate these findings in accordance with SAGER guidelines [11].

Moreover, the Shell technique presents inherent limitations as an experimental model. Although it offers mechanical stability and provides structural support for regeneration by preserving defect volume and preventing soft tissue invasion [26, 27], the procedure is technically demanding and prone to variability depending on the operator’s skill level [28]. In small animals, restricted surgical space can lead to challenges in reproducibility and stable fixation of the cortical shells. In this study, we were unable to completely standardise the volume and width of the grafted space, which may have introduced variation in the quantitative comparison among groups. Future studies may benefit from the use of 3D-printed guides or custom surgical templates to improve consistency [29].

Another consideration is the proximity of the defect to adjacent teeth, which may have influenced local bone dynamics through occlusal loading or factors related to periodontal ligaments [30]. While this represents a potential confounding variable, it also reflects one of the model’s strengths: the ability to mimic the intraoral environment, including saliva, bacterial exposure, and mechanical stress [31]. In contrast to the widely used rat model of calvarial bone regeneration, little is known about regenerative processes in the mandibular region, which holds greater clinical relevance [32]. While large-animal models such as dogs and pigs permit the creation of mandibular defects, they pose significant challenges for genetic manipulation and molecular analysis due to their high cost and procedural complexity [33]. In this context, the rat model and application of the mandibular Shell technique used in this study represents a valuable preclinical system that allows the evaluation of bone graft materials under conditions closely resembling the clinical oral environment, while also enabling molecular and genetic investigations. This model may contribute to a better understanding of material performance and biological behaviour relevant to clinical bone regeneration.

Finally, while this model replicates key intraoral conditions, surgical procedures such as stainless-steel screw fixation and trimmed cortical plates are not fully analogous to human shell augmentation. Therefore, caution is required when extrapolating these findings to clinical practice. From a clinical perspective, the present findings may help guide the selection of graft materials in bone augmentation procedures. Autogenous bone demonstrated superior osteogenic capacity and remains the gold standard when rapid and predictable bone regeneration is required. However, given its limitations, including donor site morbidity and limited graft volume, alternative materials may be considered depending on the clinical situation. OCPC showed relatively favorable regenerative potential among the synthetic and composite materials, suggesting its potential applicability in cases where autogenous bone is limited or when minimally invasive approaches are preferred. In contrast, β-TCP and AAS alone may not provide sufficient osteogenic capacity and may require combination with autogenous bone or bioactive agents to achieve clinically acceptable outcomes.Future studies are warranted to further investigate the translational relevance of these findings. In particular, studies using larger animal models, such as canine or porcine mandibular models, would better replicate clinical conditions. Moreover, longer-term evaluations are necessary to assess the stability, remodeling behavior, and long-term integration of graft materials.

## Conclusions

AB grafts produced the most bone in a rat bone graft model using the Shell technique. The findings demonstrate the superior osteogenic capacity of AB and suggest that combining autogenous bone with artificial materials may help to unlock the latent osteogenic potential of synthetic grafts. Despite some technical limitations, this model effectively replicates intraoral conditions and serves as a valuable platform for evaluating bone regeneration in clinically relevant settings.

## Data Availability

The datasets used and/or analysed during the current study are available from the corresponding author on reasonable request.

## Abbreviations

AAS: Atelocollagen absorbable sponge
AB: Autogenous bone
ANOVA: Analysis of variance
BV: Bone volume
β-TCP: β-tricalcium phosphate
EDTA: Ethylenediaminetetraacetic acid
OCPC: Octacalcium phosphate collagen composite
PBS: Phosphate-buffered saline
SD: Standard deviation
